# Transcripts involved in mitochondrial energetics are associated with VO2peak in older adults

**DOI:** 10.1093/geroni/igaf122.1853

**Published:** 2025-12-31

**Authors:** Bumsoo Ahn, Gregory Tranah, Tyler Mansfield, Paul Coen, Russell Hepple, Steven Cummings, Stephen Kritchevsky

**Affiliations:** Atrium Health Wake Forest University School of Medicine, Winston-Salem, North Carolina, United States; California Pacific Medical Center Research Institute, San Francisco, California, United States; California Pacific Medical Center Research Institute, San Francisco, California, United States; AdventHealth, Orlando, Florida, United States; University of Florida, Gainesville, Florida, United States; California Pacific Medical Center Research Institute, San Francisco, California, United States; Wake Forest University School of Medicine, Winston-Salem, North Carolina, United States

## Abstract

Abstract Mitochondrial dysfunction plays an important role in age-related mobility disability. The study of Muscle, Mobility and Aging (SOMMA) is a cohort study designed to identify muscle characteristics associated with worsening mobility. Using vastus lateralis muscle biopsies (n = 724; ages 70+ years; 56% women), we performed bulk RNAseq and used DESeq2 to test whether the expression of preselected muscle RNA transcripts related to mitochondrial energetics were associated with physical traits including cardiorespiratory fitness (VO2peak), leg power, and 400 m walk speed. In our analysis of 288 transcripts from both nuclear and mitochondria-encoded genes, several transcripts were strongly and positively associated with VO2peak. Among the top 20 differentially regulated genes (DEGs, adjusted p < 0.05) were genes involved in oxidative phosphorylation: IDH2, FH, SUCLG1 (Krebs cycle), NDUFA8 and NDUFA9 (complex I), SDHB and SDHC (complex II), CYC1 (complex III), COX5A (complex IV), and ATP5F1B, ATP5F1C, ATP5MC1, ATP5MC3, ATP5PB, and ATP5F1A (complex V). All mitochondrial DNA encoded OXPHOS complex transcripts were significantly associated with VO2peak and mitochondrial energetics. Among the genes we tested, a smaller proportion of the selected transcripts reached statistical significance (adjusted p < 0.05) for leg power and walking speed, with the highest proportion observed for VO_2_peak: VO_2_peak (66%), leg power (29%), and walking speed (11%). In summary, transcripts involved in mitochondrial energetics show a strong association with VO2peak, a trait highly dependent on mitochondrial function.

